# RETRACTED: Dal Monte et al. Fatty Acids Dietary Supplements Exert Anti-Inflammatory Action and Limit Ganglion Cell Degeneration in the Retina of the EAE Mouse Model of Multiple Sclerosis. *Nutrients* 2018, *10*, 325

**DOI:** 10.3390/nu18132171

**Published:** 2026-07-03

**Authors:** Massimo Dal Monte, Maurizio Cammalleri, Filippo Locri, Rosario Amato, Stefania Marsili, Dario Rusciano, Paola Bagnoli

**Affiliations:** 1 Department of Biology, University of Pisa, Via San Zeno 31, 56127 Pisa, Italy; maurizio.cammalleri@unipi.it (M.C.); filippo.locri@student.unisi.it (F.L.); rosario.amato@student.unisi.it (R.A.); marsili@temple.edu (S.M.); 2 Interdepartmental Research Center Nutrafood “Nutraceuticals and Food for Health”, University of Pisa, Via del Borghetto 80, 56124 Pisa, Italy; 3 Sooft Italia SpA, Contrada Molino 17, 63833 Montegiorgio (F.M.), Italy; dario.rusciano@sooft.it

The journal retracts the article titled “Fatty acids dietary supplement exerts anti-inflammatory action and limits ganglion cell degeneration in the retina of the EAE mouse model of multiple sclerosis” [1], cited above.

Following publication, concerns were raised with the Editorial Office regarding the integrity of a figure presented in this article [1].

In accordance with the journal’s standard procedures, the Editorial Office and Editorial Board conducted an investigation, that identified indications of inappropriate editing and duplication in Figure 5. Although the authors cooperated with the investigation, the explanations and supporting materials provided were not deemed sufficient by the Editorial Board to resolve the identified concerns. As a result, the Editorial Board has lost confidence in the reliability of the findings and has decided to retract this publication [1], as per MDPI’s retraction policy.

This retraction was approved by the Editor-in-Chief of *Nutrients*.

The authors have been informed of this retraction and have indicated their agreement with this decision.
